# Cocaine Administration and Its Abstinence Conditions Modulate Neuroglia

**DOI:** 10.3390/ijms21217970

**Published:** 2020-10-27

**Authors:** Kinga Gawlińska, Małgorzata Frankowska, Dawid Gawliński, Marcin Piechota, Michał Korostyński, Małgorzata Filip

**Affiliations:** 1 Maj Institute of Pharmacology Polish Academy of Sciences, Department of Drug Addiction Pharmacology, Smętna 12 Street, 31-343 Kraków, Poland; kingaw@if-pan.krakow.pl (K.G.); gawlin@if-pan.krakow.pl (D.G.); mal.fil@if-pan.krakow.pl (M.F.); 2 Maj Institute of Pharmacology Polish Academy of Sciences, Department of Molecular Neuropharmacology, Smętna 12 Street, 31-343 Kraków, Poland; marpiech@if-pan.krakow.pl (M.P.); michkor@if-pan.krakow.pl (M.K.)

**Keywords:** cocaine self-administration, extinction training, hippocampus, microglia, MYRF, oligodendroglia, PLEK, striatum

## Abstract

Cocaine induces neuronal changes as well as non-neuronal (astrocytes, microglia, oligodendroglia) mechanisms, but these changes can also be modulated by various types of drug abstinence. Due to the very complex and still incompletely understood nature of cocaine use disorder, understanding of the mechanisms involved in addictive behavior is necessary to further search for effective pharmacotherapy of this disease. The aim of this study was to investigate changes at the gene and protein levels associated with glial cell activity after cocaine exposure, as well as during early cocaine abstinence (3 days) with extinction training or in home cage isolation. Cocaine self-administration significantly decreased myelin regulatory factor (MYRF) and cyclic nucleotide phosphodiesterase (CNP) expression in the hippocampus as well as pleckstrin (PLEK) and T-lymphocyte activation antigen (CD86) in the rat striatum. Depending on cocaine abstinence conditions, microglial PLEK expression was increased through extinction training but did not change in the home cage isolation. In addition, downregulation of gene expression associated with oligodendrocytes (CNP, MYRF) and microglia regulator of G protein signaling 1 (RGS1) was observed in the hippocampus, regardless of the type of drug abstinence, while downregulation of myelin and lymphocyte protein (MAL) expression was found only in rats exposed to abstinence in the home cage. Taken together, the presented results strongly suggest that cocaine abstinence evokes significant changes in gene expression associated with the proper functioning of glial cells, suggesting their significant involvement in adaptive changes in the brain associated with cocaine exposure. Interestingly, drug abstinence conditions are important factors influencing observed changes at the transcript levels of selected genes, which may be of clinical interest.

## 1. Introduction

Substance use disorder (SUD), which is a serious threat to public health, is a multifaceted disorder. SUD, including cocaine use disorder (CUD), is defined as a chronic and complex disorder of the central nervous system, characterized by compulsive search and intake of addictive substances as a result of loss of control over the behavior [1,2,3]. According to the World Drug Report, around 19 million people are addicted to cocaine [4]. As many as 40–60% of patients in therapy due to cocaine or alcohol return to addiction within the first year. Therefore, studying adaptive changes in the brain under various abstinence conditions can contribute to a better understanding of the role of the environment that can contribute to relapses. 

Due to the very complex nature of CUD, understanding of the molecular mechanisms involved in addictive behavior and alterations in gene expression underlying behavioral dysregulation associated with this disorder appears to be crucial for the development of effective therapy [5]. The molecular substrates underlying CUD have been studied for many years, with particular emphasis on signaling molecules involved in the modulation of neural communication [6]. SUD, including cocaine, is considered a drug-induced neuroplasticity disorder [7]. Despite the key role of neurons involved in the pathophysiology of SUD, these cells are not the only nervous system component responsible for sustaining and regulating neurotransmission. Non-neuronal cells, especially astrocytes and oligodendrocytes, have been shown to play an important role in regulating neurotransmission, conducting nerve impulses, metabolizing neurotransmitters, and supplying energy metabolites to synaptic functions [8]. Evidence indicates altered expression of oligodendroglia-related genes in several brain structures from clinically diverse groups of subjects, including cocaine users [9]. Moreover, microglial cells, not directly participating in the modulation of neurotransmission, express numerous molecules associated with inflammation that become activated by neuronal injury or degeneration and then also express multiple receptors for neuropeptides and neurotransmitters capable of releasing trophic factors that are important in the survival of neurons [10,11]. The latter cells are activated following chronic cocaine treatment, leading to inflammatory responses in animal models [12] and in clinical studies [13].

In light of the above information, using an animal model of intravenous cocaine self-administration in rats, we investigated the effects of chronic cocaine self-administration and various abstinence conditions on changes in neuroglial genes in selected brain regions related to the reward system, i.e., the prefrontal cortex, striatum, and hippocampus. For this purpose, we performed gene expression analysis for selected top 20 cell-type-specific markers for the astrocytes, oligodendrocytes, and microglia. Next, we validated selected genes associated with microglia: cluster of differentiation 86 (*Cd86*), pleckstrin (*Plek*), P2Y purinoceptor 13 (*P2ry13*), and regulator of G-protein signaling 1 (*Rgs1*), as well as the following oligodendrocytes: cyclic nucleotide phosphodiesterase (*Cnp*), myelin and lymphocyte (*Mal*), and myelin transcription factor (*Myrf*). Moreover, using enzyme-linked immunosorbent assays (ELISAs), we assessed striatal pleckstrin (PLEK) and hippocampal myelin regulatory factor (MYRF) on the protein level.

## 2. Results

### 2.1. Influence of Cocaine on Transcript Levels

**Figure 1:**
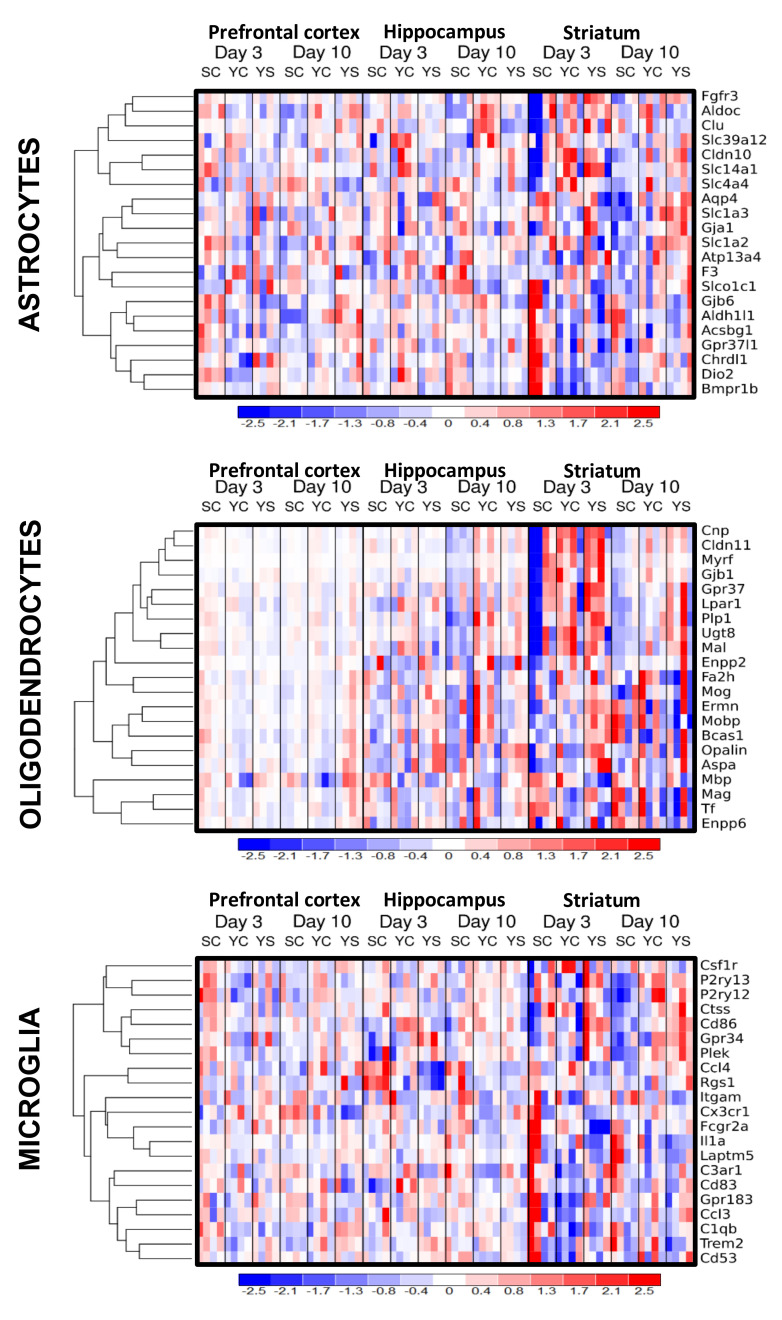
The expression of selected genes associated with astrocytes, oligodendrocytes, and microglia in the rat prefrontal cortex, hippocampus, and striatum on the 3rd and 10th days of cocaine abstinence with extinction training. The intensity of the color is proportional to the standardized values from each microarray, as displayed on the bar below the heat map images. SC, cocaine self-administration group; YC, yoked cocaine group; YS, yoked saline group (control).

Microarray analysis was conducted to evaluate the prefrontal, hippocampal, and striatal transcriptome on the short-term (3rd) and long-term (10th) day of extinction training following cocaine self-administration and yoked passive cocaine and saline administration (Figure 1). The top 20 cell-type-specific markers for astrocytes, oligodendrocytes, and microglia were identified on the basis of literature and analyzed [14]. The most significant changes in transcript levels were observed on the third day of cocaine abstinence within the striatum and the hippocampus, and thus we focused on the effects of the early abstinence period. There were significant changes in selected transcript levels associated with oligodendroglia, such as: *Mal* (F(2, 9) = 8.84, *p* < 0.01), *Myrf* (F(2, 9) = 18.45, *p* < 0.001), and *Cnp* (F(2, 9) = 8.51, *p* < 0.01) within the hippocampus, but not changes within the prefrontal cortex and striatum. Post hoc tests showed significant decreases of transcript levels for *Mal* (*p* < 0.01) and *Cnp* (*p* < 0.05), as well as a tendency for *Myrf* (*p* = 0.056) in cocaine self-administered rats versus yoked saline control. Moreover, the transcript levels associated with microglia changed: *Cd86* (F(2, 9) = 16.65, *p* < 0.001), *Plek* (F(2, 9) = 16.94, *p* < 0.001), and *P2ry13* (F(2, 9) = 23.96, *p* < 0.001) in the striatum. Post hoc analysis showed a significantly decreased level of *Cd86* (*p* < 0.01), *Plek* (*p* < 0.05), and *P2ry13* (*p* < 0.01). See Table S1 for detailed results.

### 2.2. Alterations in mRNA Expression Induced by Cocaine Self-Administration and Abstinence Conditions

**Figure 2:**
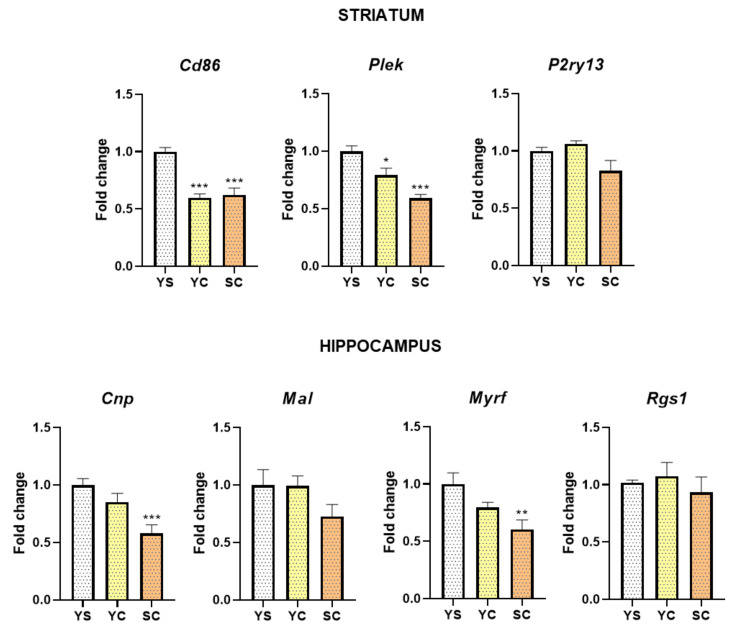
The mRNA expression levels of cluster of differentiation 86 (*Cd86*), pleckstrin (*Plek)*, and P2Y purinoceptor 13 (*P2ry13*) in the striatum and cyclic nucleotide phosphodiesterase (*Cnp*), myelin and lymphocyte (*Mal*), myelin transcription factor (*Myrf*), and regulator of G-protein signaling 1 (*Rgs1*) in the hippocampus after chronic cocaine self-administration. Number of animals was 7–8 per experimental group. Significance was determined using one-way ANOVA and the post hoc Dunnett’s test. * *p* < 0.05, ** *p* < 0.01, *** *p* < 0.001 versus yoked saline (YS) group. SC, cocaine self-administration group; YC, yoked cocaine group.

**Figure 3:**
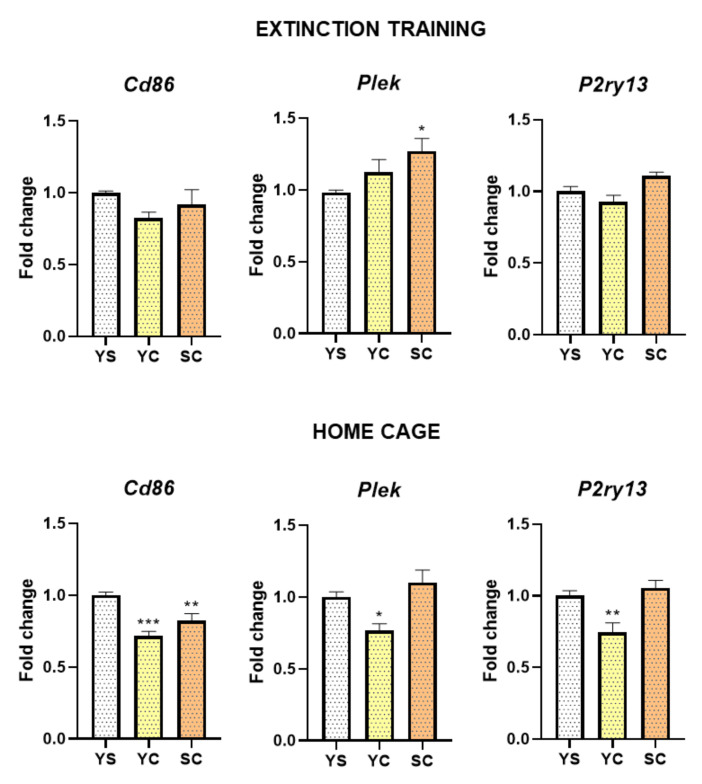
The mRNA expression levels of *Cd86*, *Plek*, and *P2ry13* in the striatum after 3 days of cocaine abstinence in different conditions (extinction training or home cage). Number of animals was 7–8 per experimental group. Significance was determined using one-way ANOVA and the post hoc Dunnett’s test. * *p* < 0.05, ** *p* < 0.01, *** *p* < 0.001 versus yoked saline (YS) group. SC, cocaine self-administration group; YC, yoked cocaine group.

**Figure 4:**
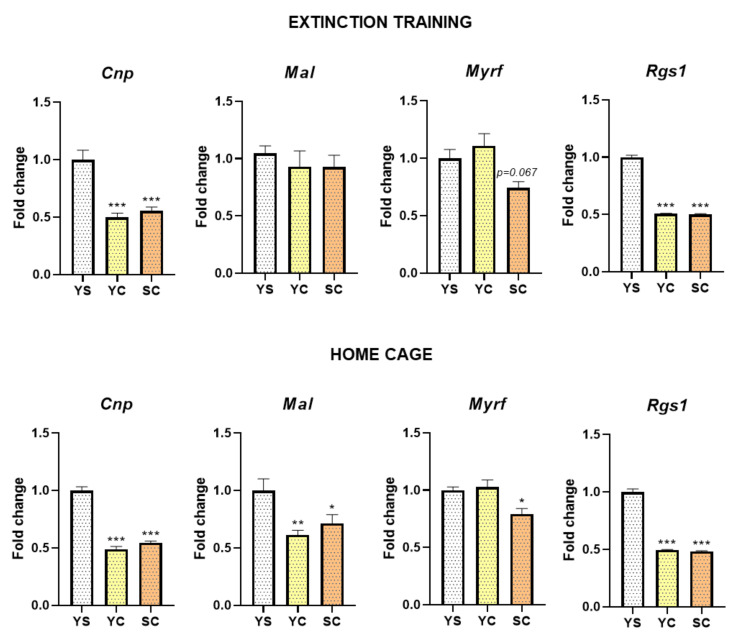
The changes in expression levels of *Cnp*, *Mal*, *Myrf*, and *Rgs1* in the hippocampus after 3 days of cocaine abstinence in different conditions (extinction training or home cage). Number of animals was 7–8 per experimental group. Significance was determined using one-way ANOVA and the post hoc Dunnett’s test. * *p* < 0.05, ** *p* < 0.01, *** *p* < 0.001 versus yoked saline (YS) group. SC, cocaine self-administration group; YC, yoked cocaine group.

Figure 2 shows the expression levels of selected genes following cocaine self-administration, while Figure 3 and Figure 4 show cocaine abstinence (3rd day) in different conditions validated using the RT-qPCR. One-way ANOVA demonstrated the significant differences in the striatal expression of *Cd86* (F(2, 18) = 25.30, *p* < 0.001) and *Plek* (F(2, 18) = 18.39, *p* < 0.001), as well as in the hippocampal expression of *Cnp* (F(2, 18) = 9.39, *p* < 0.01) and *Myrf* (F(2, 18) = 6.34, *p* < 0.01) following cocaine self-administration (Figure 2). In the striatum, we showed decreased mRNA abundance levels of *Cd86* (*p* < 0.001) and *Plek* (*p* < 0.05) in both cocaine groups. Moreover, hippocampal downregulation of mRNA expression of *Cnp* (*p* < 0.001) and *Myrf* (*p* < 0.01) following the cocaine self-administration group was observed. 

In rats exposed to abstinence in home cage isolation, statistical analysis showed significant alternations in *Cd86* (F(2, 20) = 14.66, *p* < 0.001), *Plek* (F(2, 20) = 7.25, *p* < 0.01), and *P2ry13* (F(2, 20) = 9.54, *p* < 0.01) expression level and in extinction training (3rd day) for *Plek* (F(2, 21) = 3.76, *p* < 0.05) and *P2ry13* (F(2, 21) = 6.18, *p* < 0.01). We noted an increased mRNA level of *Plek* (*p* < 0.05) in the striatum of rats self-administering cocaine following extinction training, while a decreased expression level of *Plek* (*p* < 0.05) and *P2ry13* (*p* < 0.01) in the yoked cocaine group following home cage isolation. Moreover, decreased expression of *Cd86* (*p* < 0.01) in both groups (cocaine self-administration and yoked cocaine delivery) during early (3rd day) abstinence in home cage was observed (Figure 3).

Within the hippocampus, during 3-day abstinence of cocaine self-administration, changes in expression of genes associated with the activity of oligodendrocytes (*Cnp*, *Mal*, and *Myrf1*) and microglia (*Rgs1*) were noted (Figure 4). Statistical analyses showed significant differences in *Cnp* (F(2, 21) = 130.60, *p* < 0.001), *Mal* (F(2, 21) = 6.85, *p* < 0.01), *Myrf* (F(2, 21) = 7.21, *p* < 0.01), and *Rgs1* (F(2, 21) = 379.40, *p* < 0.001) gene expression in home cage isolation, as well as in *Cnp* (F(2, 21) = 24.06, *p* < 0.001), *Myrf* (F(2, 21) = 5.29, *p* < 0.05), and *Rgs1* (F(2, 21) = 613.30, *p* < 0.001) gene expression in rats during cocaine abstinence under extinction training. A significant reduction in mRNA expression of *Cnp* (*p* < 0.001) and *Rgs1* (*p* < 0.001) was found in rats after cocaine administration, following both extinction training and home cage isolation in the hippocampus. During early cocaine abstinence, a significant decrease in *Mal* (*p* < 0.05 for cocaine self-administered group, *p* < 0.01 for yoked cocaine group) and *Myrf* (*p* < 0.05 for cocaine self-administered group) mRNA levels was seen after home cage isolation.

### 2.3. Alterations in MYRF and PLEK Protein Levels Induced by Cocaine Self-Administration and Abstinence Conditions

**Figure 5:**
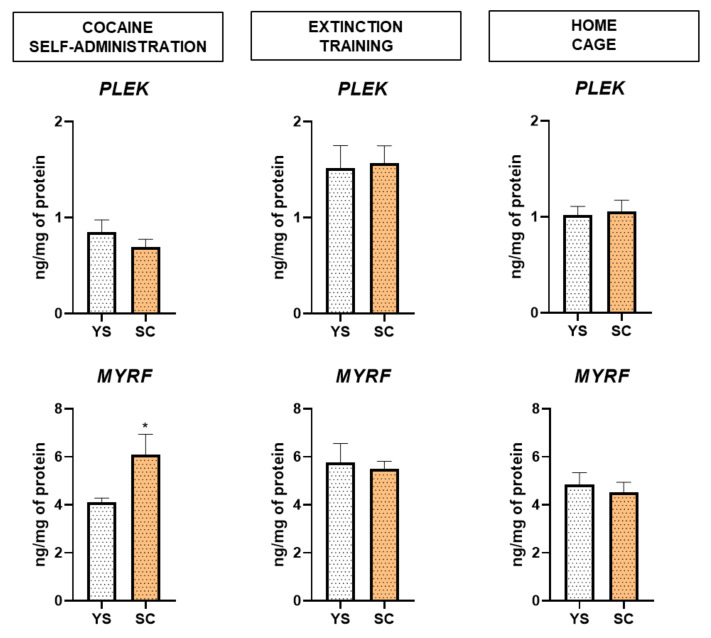
Effects of cocaine self-administration and abstinence conditions (3rd day) on striatal pleckstrin (PLEK) and hippocampal myelin regulatory factor (MYRF) protein levels. The results are expressed as the mean (± SEM). *N* = 6 rats per group. Data were analyzed using Student’s *t*-test. * *p* < 0.05, versus yoked saline (YS) group. SC, cocaine self-administration group.

Figure 5 shows that during cocaine self-administration, the MYRF protein level was significantly increased in rats (t = 2.254, df = 10, *p* < 0.05). The abstinence conditions had no effect on the examined protein levels. The PLEK protein level did not reach statistical significance.

## 3. Discussion

Uncovering the mechanism and regulation of cocaine reward and abstinence will aid in a better understanding of CUD. In this study, we performed transcript and protein analyses to evaluate the influence of cocaine self-administration and its different abstinence conditions (abstinence with extinction training or isolation in home cages) on alternation in genes associated with the proper functioning of glial cells in the selected limbic structures. We focused on the prefrontal cortex, striatum, and hippocampus – brain areas involved in habit learning, addictions, and compulsive drug-seeking behaviors [15].

The main observation of the present study is that cocaine self-administration and its abstinence conditions as well as the type of this psychostimulant intake (self-administering cocaine or yoked cocaine delivery) modulate neuroglia in the rat hippocampus and striatum. After cocaine self-administration, we found reduced mRNA expression of *Cnp* and *Myrf* in the hippocampus and *Plek* in the striatum in the cocaine self-administrated group, while cocaine administration led to downregulation of striatal *Cd86* expression in rats. Then, we examined the effect of abstinence conditions. Early extinction training and abstinence in home cages in rats with a history of cocaine self-administration induced the downregulation of hippocampal *Cnp*, *Myrf*, and *Rgs1* expression. Furthermore, the downregulation expression of *Mal* was observed only during home cage isolation. At the protein level, we found an increased hippocampal MYRF level after cocaine self-administration. No significant change was noticed for the PLEK protein level. Further, any significant astrocyte-associated gene was found to be related with short-term cocaine abstinence. 

In the present paper, significant changes in some oligodendrocyte-derived master regulators of the myelin gene regulatory network were observed. Myelin is a lipid-rich, spiraled membrane structure composed of (1) tightly packed spiraling layers of membrane that lack cytoplasm, called compact myelin, and (2) cytoplasmic noncompact regions connecting the oligodendrocyte cell body to the axonal side of the wraps and the nodes of Ranvier (for a review, see [16]). Thus, we found a reduction in *Myrf* transcript resulting from cocaine reward that lasted even up to the 10th day of the drug-free period and regardless of the type of cocaine abstinence in the rat hippocampus. At the same time, cocaine motivational properties developed during successful learning in the self-administration task increased the MYRF protein level. MYRF is entirely expressed in differentiated oligodendrocytes [17], where it is critical for myelination in a developing nervous system, while in the adult central nervous system it is required for oligodendrocyte generation, and its conditional deletion leads to defeats in motor learning [18]. Further, in the hippocampus, we observed changes in *Mal* expression, i.e., a trend of inhibition in cocaine self-administering rats and a reduction in isolation during cocaine abstinence for cocaine-treated animals. MAL is a transmembrane–tetraspan proteolipid that is a part of compact myelin and expressed by oligodendrocytes and Schwann cells [19]. Moreover, it acts as a co-activator of serum response factor (SRF) and interacts with G-actin or F-actin proteins that form dendritic spines. Regarding the latter mechanism, it was recently found that cocaine-induced SRF–MAL protein expression in the nucleus accumbens is essential for the cocaine contingent (self-administered) and non-contingent delivery for spine morphogenesis and behavioral sensitization [20]. Next, the latter authors demonstrated that (1) on the basis of the duration of cocaine withdrawal, the increase (1 day) and reduction (30 day) in MAL was localized to the crude nuclear, but not cytoplasm, fraction, as well as (2) the role of glutamatergic transmission from medium prefrontal cortex inputs to the nucleus accumbens (but not the ventral hippocampus–nucleus accumbens pathway) in accumbal SRF–MAL protein expression. The above findings seem to limit the possibility that inhibition of hippocampal *Mal* expression as shown in the present study is linked to *Mal* alterations in the nucleus accumbens [20]. Furthermore, downregulation in the hippocampal *Mal* expression (present study) is also not related to impulsivity—as this kind of behavior is a key component of CUD [21]—since increased accumbal *Mal* in highly impulsive rats was recently discovered [22].

Another studied oligodendrocyte gene was *Cnp*, a transcript linked with non-compact myelin sheaths and engaged in their maintenance and function [23]. Here, we observed a reduction in hippocampal *Cnp* expression during cocaine self-administration and during drug-free periods in any abstinence conditions. These adaptive changes reflect specific cocaine-mediated changes but not toxic effects of the drug on oligodendrocytes since (1) the changes were not present in the prefrontal cortex or striatum and (2) expression on the other myelin-related genes was not altered. Available postmortem data from drug abusers showed no changes in *Cnp* transcript expression in striatal areas in cocaine-abusing subjects while showing marked decreases in the expression of mRNAs for other myelin-related proteins in the striatal areas [24,25,26]. Of note, alcoholic cases resulted in lower *Cnp* expression in the frontal cortex [27]. 

Our findings demonstrate that cocaine induced selective reprogramming of myelin gene expression in the rat hippocampus (but not in the prefrontal cortex or striatum). Some laboratories using different cocaine treatments, behavioral protocols, or animal strain demonstrated reduction of the density of oligodendrocyte and/or myelin-associated genes following 2-month withdrawal from extended (6 h) cocaine self-administration in rat cortices or following cocaine non-contingent administration in mice striatal areas [28,29]. The disturbances of oligodendrocyte affected by SUD may be important because deficits of myelination in oligodendrocytes due to loss of ErbB signaling are concomitant with increases in dopamine signaling components (receptors and transporters) [30], emphasizing the relationship between the myelination process and dopaminergic neurotransmission in reward circuits. 

The hippocampus is the key structure responsible for mnemonic functions including procedural memory, a type of memory necessary for motor sequence learning ([31,32,33], but see also [34,35]), and myelin plays a role in this functional event as it allows for rapid propagation of action potentials through axons. Since difficulties were demonstrated in learning and psychomotor functions even in short-term cocaine users [36], cocaine-induced hippocampal oligodendrocyte abnormalities can contribute to the cognitive, motor, and psychological deficits that often afflict cocaine abusers. It should also be underlined that the hippocampus is linked to depressive behaviors (which appear during cocaine abstinence), and oligodendrocytes are also able to secrete neurotrophic factors in response to surrounding neurons [37]. As has been shown, oligodendrocyte impairment reduces myelination and disturbs the expression of myelin-associated genes in both SUD and depression [38]. Since there are no studies showing directly that defects in the spatial learning and memory ability and/or depressive phenotype of cocaine-treated rats are related to myelin damage in the hippocampus and that hippocampal myelin damage repair can improve the above behaviors, directly testing mechanisms responsible for these changes is an interesting avenue for further exploration. 

Cocaine activates microglia both in vitro and in vivo [39]. Our next important findings indicate reduced expression of top markers for microglia in the striatum, such as *Cd86*, *Plek*, and *P2ry13* after cocaine self-administration. The fall lasted up to the third day of abstinence under conditions of social isolation (home cage), while extinction training restores baseline levels of *Cd86* and *P2ry13*, leading to an increase (perhaps compensatory) of *Plek* expression. Apart from changes in striatal microglial cells, we also report a significant reduction in the hippocampal expression of *Rgs1* following cocaine exposure in rats, regardless of the type of drug delivery (self-administration, yoked cocaine) and drug abstinence (extinction training, isolation). 

The mechanism related to the above reduction in microglia-linked transcripts may be linked with increased apoptosis of microglial cells as a result of chronic exposure to cocaine. In fact, microglia are sentinels of the brain that are capable of releasing trophic factors affecting neuronal activity and are directly involved in the function of repairing and removing debris after brain damage [40]. Microglia cells, like other cells of the immune system line, show functional opioid receptors [41]. It is known that chronic exposure to cocaine indirectly leads to changes in the expression of opioid receptors [42]. Excessive stimulation of these receptors can promote apoptosis of microglia [43]. Interestingly, during extinction training, only after cocaine self-administration (not in yoked cocaine delivery) was the expression of *Plek* upregulated in rats, which links cocaine-related motivational processes and the change in the observed transcript. In addition, in animals exposed to abstinence in the home cage, we noted a reduction in the expression of *Plek*, *Cd86*, and *P2ry13* genes, which confirms that cocaine abstinence conditions can lead to different neurochemical changes in the brain and, as a consequence, are key to the treatment of CUD in humans.

In the literature, there are limited data focusing on the impact of cocaine on selected gene expression associated with microglia activity [44,45]. There are studies that indicate the participation of PLEK in multiple sclerosis, which is a chronic autoimmune demyelinating disease of the central nervous system. This disease is suggested to be caused by environmental factors in individuals who are genetically susceptible, leading to the activation of autoreactive T cells [46]. 

P2Y receptors play a key role in the physiology and pathophysiology of diseases such as epilepsy, depression, Parkinson’s disease, and Alzheimer’s disease [47]. These are interesting targets for pharmacotherapy, including alterations in platelet aggregation and inflammation [48]. On the basis of the available literature, the functional significance of downregulation of *P2ry13* expression in the yoked cocaine delivery group exposed to drug abstinence in home cages is nameless. 

Significant decreases in *Plek* and *P2ry13* expression only in yoked cocaine rats following home cage isolation may also be a marker of mood disturbances that occurred in those animals. In fact, stress is considered an essential reaction for yoked animals, and this aversive procedure enhances corticosterone levels and reduces the motivational aspect of cocaine [49]. However, the understanding of *Plek* and *P2ry13* alterations in cocaine abstinence needs to be investigated in prospect studies.

CD86 is expressed on monocytes and plays a key role in lymphocyte activation and, thus, adaptive immunity [50]. In the present study, the striatal level of *Cd86* was significantly reduced, both in cocaine self-administrated and yoked cocaine delivery rats only after abstinence in a home cage. These changes cannot be linked with cocaine pharmacological or toxicological effects, as gene expression was on the control level in rats early extinguished from cocaine self-administration. Furthermore, 3 days following discontinuation of cocaine self-administration, we found a significant increase in immobility, with time being a marker for the depressive phenotype [51]. Whether a reduction in the *Cd86* transcript is altered by depression or any emotional states dependent on cocaine abstinence in home cages requires further analyses. 

RGS1 is a cytosolic protein linked with regulation of G-protein signaling. Its haplotype is associated with—among others—depression and anxiety [52] and schizophrenia [53]. Since these psychiatric symptoms are associated with cocaine abstinence, reduction in hippocampal *Rgs1* may be a signaling marker corresponding to such phenotypes.

Taken together, the present data suggest that cocaine abstinence with extinction training and abstinence in the home cage significantly affects microglia and oligodendrocytes at the level of gene expression. Moreover, our data indicate that expression levels of key glia-related genes in the hippocampus (*Mal*) and striatum (*Cd86*, *Plek*, *P2ry13*) vary depending on the type of abstinence from cocaine. Neuroglia activation can result in phenotypic and functional diversity. However, the pathways that trigger different states of microglial activation remain to be fully understood. Cocaine induces neuronal changes as well as non-neuronal mechanisms, but these changes can also be modulated by various types of abstinence. Hopefully, future research will lead to a deeper understanding of the mechanisms involved in the effects of cocaine that could help to discover new targets in drug design and effective SUD pharmacotherapy.

## 4. Materials and Methods

### 4.1. Animals

Male Wistar rats (*n* = 118, 290–350 g; Charles River Laboratories, Sulzfeld, Germany) were used for the study, in accordance with the European Directive 2010/63/EU and with approval from the Local Ethics Commission at the Maj Institute of Pharmacology Polish Academy of Sciences (901/2012; 967/2012; 148/2016; 173/2016). Animals were housed in standard conditions—in home cages (5 rats per cage during training or individually after surgery), 22 ± 2 °C, and at 55 ± 10% humidity, with 12 h (lights on at 6:00 a.m.) light–dark cycles. Animals had free access to food (Labofeed pellets) and water, excluding a period of initial lever press training and a day with retraining following surgery where water was limited for 2 h/day after a 2 h water training session. All efforts was made to minimize suffering and the number of animals used.

### 4.2. Cocaine Self-Administration and Extinction Training

The initial training, surgical, and cocaine self-administration procedures used have been described previously [54,55,56]. Before implantation with indwelling jugular catheters, animals underwent initial lever press training for water reinforcement for 1 week with increasing fixed ratio (FR) requirements (FR1; FR3; and finally, FR5). The catheters were flushed every day alternately with 0.2 mL of an antibiotic solution of cefazolin (10 mg/Kg; Tarfazolin, Polfa, Warszawa, Poland) dissolved in heparinized saline (70 U/mL in 0.9% sterile saline: Polfa, Warszawa, Poland) or with heparinized saline. After recovery (at least 7 days), the rats were randomly assigned to either cocaine self-administration (*n* = 12–14/group) or a yoked group (cocaine or saline; *n* = 8/group). Then, animals were trained to perform cocaine self-administration in operant chambers as previously described [53,54,55]. The experiments were conducted in standard operant chambers (Med Associates, Fairfax, VT, USA), which contain a retractable two lever (active and inactive), an exhaust fan, a house light, a stimulus light directly above the retractable lever, and a tone source (2000 Hz). For self-administering rats, presses on the cocaine-paired lever (FR5) resulted in an intravenous delivery of 0.5 mg/Kg cocaine (cocaine HCl; Sigma-Aldrich, St. Louis, MO, USA) in 0.1 mL sterile 0.9% NaCl infusion paired with the conditioning light and tone cue over 5 s (inactive lever presses had no consequence). Each infusion was followed by a 20 s time-out period. Rats in the experiment underwent a minimum 12-day session (2 h daily sessions 6 days/week) and acquired the self-administration criterion (a 3-day period during maintenance in which the number of active lever presses varied by 10% or less). The experimental events were scheduled, and data collection was controlled via computer with Med Associates interface and software (Med-PC IV software, MED Associates, Fairfax, VT, USA). 

Following the last cocaine self-administration session, the rats performed lever pressing in the same operant chambers, which lasted for 2 h daily; during extinction training, cocaine infusion or presentation of the drug-paired cues was withheld. Instead, saline (0.1 mL/infusion) was delivered. During abstinence in the home cage condition, to reduce social interactions, the animals lived individually in the plastic cage with white walls in a room to which only the experimenter had access, and animals were handled once per week. 

Animals that during the recovery or self-administration period (*n* = 4 rats) had problems with the catheters or did not complete the self-administration acquisition/maintenance criterion (*n* = 18 rats) were removed from the experiments. The animals showed stable behavioral responses during cocaine self-administration (last session: active lever presses 155–157 ± 11–14; inactive lever presses 3–7 ± 1–3); the full behavioral results have been described in our previously reports in Sadakierska-Chudy et al. [56]. The animals were the same as in our previous study (*n* = 8/group).

### 4.3. Brain Isolation and RNA Extraction

The rats were sacrificed immediately after the experimental session. The prefrontal cortex, striatum, and hippocampus were rapidly dissected out according to the Rat Brain Atlas [57] and placed on dry ice and frozen at −80 °C. First, structures were homogenized (Bioprep-24 Homogenizer; Aosheng, China). The RNA/DNA/PROTEIN Purification Plus Kit (Norgen Biotek, Thorold, ON, Canada) was used to isolate RNA in accordance with the manufacturer’s instruction. The quantity of RNA was checked with a NanoDrop ND-1000 Spectrophotometer (Thermo Scientific, Wilmington, DE, USA), and the RNA integrity was analyzed with an RNA 6000 Nano Chip Kit and an Agilent Bioanalyzer (Agilent Technologies, Santa Clara, CA, USA).

### 4.4. Microarray Analyses

Gene expression was analyzed using the Rat 4x44K Gene Expression Array v2 (Agilent Technologies, Santa Clara, CA, USA). Sample labeling and hybridization were performed using the Agilent One-Color Microarray-Based Gene Expression Analysis. Four pools of RNA (RNA of two rats at equal concentrations from each experimental groups (*n* = 8/group)) containing 2 μg of total RNA were converted to complementary DNA (cDNA) and transcribed into complementary RNA (cRNA) in the presence of cyanine 3- uridine triphosphate(Cy3-UTP) Next, cRNA was hybridized for 17 h at 65 °C with rotation and washed to remove nonspecific hybridization. Microarrays were scanned using the Agilent Microarray Scanner and Feature Extraction software (v.11.0.1.1) (Agilent Technologies, Santa Clara, CA, USA). The GeneSpring GX software, v.12.1 (Agilent Technologies, Santa Clara, CA, USA), was used to normalize and analyze the data. For genes with multiple probes, we defined gene expression as an average signal of all the probes. The fold change was used to identify the differentially expressed genes compared to the yoked saline group.

The list of cell-type-specific gene expression markers was obtained from the Supplementary Material (Table S1) of recently published data [14]. The measures were based on aggregating the signatures across the combination mouse and human datasets (sheet: top_all_enrich). The top 20 most enriched genes for the astrocytes, oligodendrocytes, and microglia were selected and further analyzed.

### 4.5. Quantitative Real-Time PCR

Reversed transcription to cDNA was performed using a High-Capacity cDNA Reverse Transcription Kit (Applied Biosystems, Foster City, CA, USA). qRT-PCR was performed in duplicate on a 96-well plate using the Quant Studio 3 (Applied Biosystems, Foster City, CA, USA), and applying TaqMan Gene Expression Assays (Thermo Fisher Scientific, Waltham, MA, USA) for Mal (Rn00562993_m1), Myrf (Rn01454573_m1), Cd86 (Rn00571654_m1), Plek (Rn01429661_m1), P2ry13 (Rn02345727_s1), and Cnp (Rn01399463_m1). Conditions were as follows: an initial step of 95 °C for 10 min, followed by 40 cycles of 95 °C for 15 s and then 60 °C for 60 s. Hprt1 (Rn01527840_m1) expression was used as housekeeping control. Values are expressed as fold change from the control (yoked saline group).

### 4.6. Determination of MYRF and PLEK Protein Concentration

The hippocampal MYRF and striatal PLEK protein levels were measured using ELISA kits (cat. no. EIA07980r, EIA08179r; Xinqidi Biological Technology, Wuhan, China) in accordance with the manufacturer’s protocol. Duplicates of each sample and standards were transferred to ELISA plates. Absorbance was measured at a wavelength of λ = 450 nm using a Multiskan Spectrum spectrophotometer (Thermo LabSystems, Philadelphia, PA, USA). The concentration of proteins was calculated from a standard curve and expressed as ng/mg of protein. For protein measurement, the Pierce BCA Protein Assay Kit (Thermo Scientific, Rockford, IL, USA) was used.

### 4.7. Statistical Analyses

All data were expressed as mean ± standard error of mean (SEM). Gene expression or protein levels data for each abstinence condition and brain structure were analyzed with one-way ANOVA followed by a Dunnett’s post hoc test or Student’s *t*-test using GraphPad Prism 8.4.3 software (GraphPad Software, La Jolla, CA, USA). *P* < 0.05 was considered statistically significant.

## Supplementary Materials

Supplementary available at: https://www.mdpi.com/1422-0067/21/21/7970/s1. Table S1. Microarray analysis results.

## Abbreviations

CD86	cluster of differentiation 86
CNP	cyclic nucleotide phosphodiesterase
CUD	cocaine use disorder
MAL	myelin and lymphocyte protein
MYRF	myelin regulatory factor
PLEK	pleckstrin
P2RY13	P2Y purinoceptor 13
SUD	substance use disorder
RGS1	regulator of G protein signaling 1
